# The cell surface mucin podocalyxin regulates collective breast tumor budding

**DOI:** 10.1186/s13058-015-0670-4

**Published:** 2016-01-22

**Authors:** Marcia L. Graves, Jane A. Cipollone, Pamela Austin, Erin M. Bell, Julie S. Nielsen, C. Blake Gilks, Kelly M. McNagny, Calvin D. Roskelley

**Affiliations:** 1 Department of Cellular and Physiological Sciences, University of British Columbia, 2350 Health Sciences Mall, Vancouver, BC V6T 1Z3 Canada; 2 Deeley Cancer Research Center, 2410 Lee Avenue, Victoria, V8R 6V5 BC Canada; 3 Genetic Pathology Evaluation Center, Vancouver General Hospital, 2660 Oak Street, Vancouver, V6H 3Z6 BC Canada; 4 Biomedical Research Center, University of British Columbia, Vancouver, BC V6T 1Z3 Canada

## Abstract

**Background:**

Overexpression of the transmembrane sialomucin podocalyxin, which is known to play a role in lumen formation during polarized epithelial morphogenesis, is an independent indicator of poor prognosis in a number of epithelial cancers, including those that arise in the breast. Therefore, we set out to determine if podocalyxin plays a functional role in breast tumor progression.

**Methods:**

MCF-7 breast cancer cells, which express little endogenous podocalyxin, were stably transfected with wild type podocalyxin for forced overexpression. 4T1 mammary tumor cells, which express considerable endogenous podocalyxin, were retrovirally transduced with a short hairpin ribonucleic acid (shRNA) targeting podocalyxin for stable knockdown. In vitro, the effects of podocalyxin on collective cellular migration and invasion were assessed in two-dimensional monolayer and three-dimensional basement membrane/collagen gel culture, respectively. *In vivo*, local invasion was assessed after orthotopic transplantation in immunocompromised mice.

**Results:**

Forced overexpression of podocalyxin caused cohesive clusters of epithelial MCF-7 breast tumor cells to bud off from the primary tumor and collectively invade the stroma of the mouse mammary gland *in vivo.* This budding was not associated with any obvious changes in histoarchitecture, matrix deposition or proliferation in the primary tumour. In vitro, podocalyxin overexpression induced a collective migration of MCF-7 tumor cells in two-dimensional (2-D) monolayer culture that was dependent on the activity of the actin scaffolding protein ezrin, a cytoplasmic binding partner of podocalyxin. In three-dimensional (3-D) culture, podocalyxin overexpression induced a collective budding and invasion that was dependent on actomyosin contractility. Interestingly, the collectively invasive cell aggregates often contained expanded microlumens that were also observed *in vivo*. Conversely, when endogenous podocalyxin was removed from highly metastatic, but cohesive, 4T1 mammary tumor cells there was a decrease in collective invasion in three-dimensional culture.

**Conclusions:**

Podocalyxin is a tumor cell-intrinsic regulator of experimental collective tumor cell invasion and tumor budding.

**Electronic supplementary material:**

The online version of this article (doi:10.1186/s13058-015-0670-4) contains supplementary material, which is available to authorized users.

## Background

It is often proposed that metastatic carcinoma progression begins when single cells which have undergone an epithelial to mesenchymal transition (EMT) break away from the primary tumor mass and begin to invade the surrounding tissue stroma [1]. However, when the clinical tumor–stroma interface is examined in detail, the presence and extent of small cohesive clusters of invasive cells, often denoted as “tumor buds”, correlates with metastatic progression and poor prognosis in a number of solid tumor types [2–8], including those of the breast [6–8]. The cells within these tumor buds, which invade collectively, maintain at least a modicum of their original epithelial phenotype and they often continue to form cell junctions with their neighbors [9]. While collective tumor cell invasion has been extensively modeled experimentally [10], clinically-relevant drivers of the process are only now beginning to be identified [11, 12].

Podocalyxin is a transmembrane sialomucin that is normally localized to the apical surface of a variety of epithelia, including the luminal cells of breast ducts and lobules [13]. The physiologic function of podocalyxin was initially characterized in the developing kidney where the anti-adhesive properties of the mucinous extracellular domain act coordinately with the actin cytoskeleton-associated cytoplasmic domain to physically separate apical membrane processes between neighboring glomerular epithelial cells during the formation of the primary urinary filter [14, 15]. Podocalyxin also plays a more general role in directing the morphogenesis of tubular and glandular epithelia by facilitating the formation of nonadhesive apical membrane domains during lumen formation [16–18]. Interestingly, Mostov and colleagues recently demonstrated that podocalyxin can also cause normal epithelial cells to become collectively invasive in three-dimensional (3-D) culture when polarity cues are actively disrupted such that apical membrane domains and lumens do not form efficiently [19].

Podocalyxin is highly expressed in a number of human tumors, most of which are epithelially derived, with varying degrees of prognostic significance [20–33]. In the case of invasive breast cancer, podocalyxin overexpression is an independent marker of poor outcome [13]. At the cellular level, podocalyxin overexpression expands apical membrane domains on the free surface of epithelial breast cancer cells that are maintained in monolayer culture where it alters the subcellular localization of two associated actin-binding scaffolding proteins, NHERF-1 and ezrin [34], both of which have been implicated in breast tumor progression when they are mistargeted [35–37]. Importantly, Casey and colleagues demonstrated that a podocalyxin–ezrin complex increases breast tumor motility [38]. These findings, coupled with the recent observation that podocalyxin overexpression is positively correlated with lymphovascular invasion (LVI) in breast cancer [31], which itself is well correlated with tumor budding [8], led us to ask whether podocalyxin overexpression plays a functional role in collective breast tumor cell invasion.

We found that podocalyxin overexpression caused epithelial MCF-7 breast tumor cell-derived xenotransplants to generate cohesive micronodules that budded from the primary tumor and collectively invaded the stroma of the mammary fat pad of immunocompromised mice. In two-dimensional (2-D) monolayer culture, podocalyxin overexpression induced a collective MCF-7 cell migration that was ezrin dependent. In addition, in 3-D culture, podocalyxin overexpression caused cohesive MCF-7 tumor cell clusters to expand their poorly formed internal microlumens and induced a collective invasion that was dependent on actomyosin contractility. Conversely, the stable downregulation of podocalyxin attenuated the collective invasion of epithelial, but highly metastatic, 4T1 mammary tumor cells. Therefore, podocalyxin is a clinically-relevant marker of breast cancer progression that induces collective tumor cell motility and invasion as well as experimental tumor budding when it is highly expressed in epithelial breast tumor cells.

## Methods

### Cell lines

Human MCF-7 breast cancer cells were purchased from ATCC (Manassas, VA, USA). These cells were cohesive, epithelial, and estrogen dependent for growth in vivo. Mouse 4T1 mammary tumor cells were kindly provided by Dr Fred Miller (Wayne State University, Detroit, MI, USA).

### Podocalyxin overexpression and downregulation

For overexpression studies, MCF-7 cells were transfected with a pIRES2-based vector (Clonetech, Mountain View, CA, USA) without or with the full-length murine podocalyxin cDNA [34]. Pooled, genetically selected, stable cell populations were subjected to two independent rounds of fluorescent-activated cell sorting (FACS) after indirect immunofluorescent labeling of murine podocalyxin using a species-specific antibody (anti-mouse PCLP-1; MBL, Nagoya, Japan).

For downregulation studies, three different short hairpin RNA (shRNA) sequences designed for targeted knockdown of murine podocalyxin were identified using PSI Oligomaker v1.5 freeware (http://web.mit.edu/jacks-lab/protocols/pSico.html) and were cloned into the pLL3.7 lentiviral vector. Lentiviral particles were generated after transfecting 293T cells with the pLL3.7 vector and packaging plasmids (pVSVg, pRSV-Rev, pMDLgag/pol) followed by transduction into subconfluent 4T1 mouse mammary tumor cells. Infected 4T1 cells were then subjected to bulk FACS for green fluorescent protein expression. Two of the resulting cell populations demonstrated reduced podocalyxin expression, one of which was used for functional analysis (4T1-Podo-KD 3603; see Fig. 7).

To assess steady-state protein levels, cells were lysed in RIPA buffer (150 mM NaCl, 50 mM Tris pH 7.4, 5 mM ethylenediamine tetraacetic acid, 1.0 % NP-40, 0.5 % sodium deoxycholate, and 0.1 % sodium dodecyl sulfate) with protease and phosphatase inhibitors. Equal amounts of protein were then separated by SDS-PAGE, transferred onto PVDF membranes and probed with an anti-mouse podocalyxin primary antibody (1 μg/ml; R&D Systems, Minneapolis, MN, USA), and primary antibodies against E-cadherin (0.1 μg/ml; BD Biosciences, Mississauga, ON, Canada), ZO-1 (0.25 μg/ml; Invitrogen-Zymed Laboratories, South San Francisco, CA, USA), and epithelial cytokeratins (clones AE1/AE3; Dako, Troy, MI, USA). Primary antibody binding was visualized with the appropriate species-specific horseradish peroxidase-conjugated second antibody (Jackson Immunoresearch, West Grove, PA, USA) followed by chemoluminescence.

### Orthotopic breast tumor xenografts, quantification, and histological analysis

17β-estradiol tablets (60-day release; IRA, Sarasota, FL, USA) were implanted subcutaneously into the cervical scapular space of 12-week-old female Rag 2 M mice (Taconic, Hudson, NY, USA) and 1.0 × 10^6^ MCF-7 cells mixed 2:1 with Matrigel were innoculated into the right abdominal (#4) mammary fat pad. After 6 weeks, the mice were sacrificed and the entire mammary fat pad together with embedded tumor material was excised. Final tumor volumes were calculated using the formula:

$$ \mathrm{Final}\ \mathrm{tumor}\ \mathrm{volume} = 0.52\left(\mathrm{length}\ \left(\mathrm{mm}\right)\right) \times \left(\mathrm{width}\ \left(\mathrm{mm}\right)\right) \times \left(\mathrm{height}\ \left(\mathrm{mm}\right)\right). $$

Approval for the xenograft study was obtained from the Animal Care Committee of the University of British Columbia.

Formalin-fixed and paraffin-embedded mammary glands (control, *n* = 8; podocalyxin, *n* = 7) were serial sectioned in their entirety. Every 10th section was deparaffinized and stained with hematoxylin and eosin (H & E) as well as Masson’s trichrome using standard procedures. Stained sections were qualitatively assessed for tumor morphology, tumor budding/collective microinvasion at tumor/stromal borders, invasion of tumor cells into existing normal mammary ducts, and infiltration into the surrounding vasculature and local lymph node.

Microinvasion was quantified by counting the number of tumor buds that extended into the stroma surrounding the main primary tumor. The assessed sections were selected based on the relative location of the inguinal lymph node (20 H & E-stained sections, each at least 50–100 μm apart, on either side of the lymph node were analyzed; i.e., 40 total sections per tumor through an approximate tissue depth of 2–4 mm) and the average total number of small nodules/buds and the ratio of the quantity of small nodules/buds per tumor volume are shown.

Deparaffinized tissue sections were antigen-retrieved in heated citrate buffer, blocked, and incubated with primary antibodies against E-cadherin (0.1 μg/ml; BD Biosciences), multiple epithelial cytokeratins using a broad-spectrum antibody (1:200; Dako), murine podocalyxin (1 μg/ml; R&D Systems), Ki67 (1:100; Abcam Inc., Cambridge, MA, change to: 'USA'), and ezrin (1 μg/ml; Cell Signaling Technology Inc., Danvers, MA, USA). Antibody binding was visualized using a horseradish peroxidase-labeled polymer (EnVision™ + System; Dako), developed with Nova Red™ (Vector Laboratories, Burlingame, CA, USA), and counterstained with Mayer’s hematoxylin.

### 2-D migration assays

Confluent MCF-7 cell monolayers were serum-starved overnight, scratched with a p-200 micropipette tip, and treated with 100 ng/ml epidermal growth factor (EGF) for 16 hours. For live imaging, phase micrographs were taken with a Nikon TMS phase Microscope equipped with a Nikon Coolpix digital camera (Nikon, Mississauga, ON, Canada) to capture the migratory infilling of the scratch/wound. Wound areas were quantified using CellSens Software (Olympus, Richmond Hill, ON, Canada).

In some experiments the scratched monolayers were treated with either dimethylsulfoxide (DMSO; vehicle control) or the ezrin inhibitor NSC668394 (Millipore, Etobicoke, ON, Canada [39]) diluted to 10 μM in DMSO.

### 3-D culture assay, quantification, and image analysis

MCF-7 cells in low serum media (Dulbecco’s modified Eagle’s medium/F12 with 1 % fetal bovine serum) were plated overnight onto reconstituted basement membrane gels (Matrigel™; BD Biosciences). Spheroidal cell aggregates were then overlaid with neutralized collagen I (2.5 mg/ml; BD Biosciences) and maintained in low serum media supplemented with EGF (100 ng/ml; Sigma, St Louis, MO, USA) for an additional 4 days. The myosin II inhibitor blebbistatin (10 mg/ml; Sigma) was added to the media during extracellular matrix (ECM) overlay (day 0) and again on day 2. On day 4 the tumor spheroids/elongating aggregates were fixed for immunocytochemistry and confocal microscopic analysis as described in the next section. To quantify spheroid/aggregate shape, the longest and shortest axes (length and width respectively) were measured using CellSens Software (Olympus) and expressed as an elongation index (length/width).

For time-lapse imaging, collagen I-overlaid MCF-7 cell 3-D spheroids were maintained in a stage-top incubator (Chamlide TC™; Live Cell Instrument, Seoul, Korea), and invasion was imaged live over a 24-hour period using a Leica inverted DMI 4000 phase microscope equipped with a DFC345 FX CCD camera and AF6000 imaging software (Leica Microsystems, Concord, ON, Canada).

The 3-D culture of 4T1 cells was performed as already described except that the cells were maintained in the absence of serum and EGF, given that these metastatic cells are completely growth factor-independent for motility. Collagen I-overlaid 4T1 cultures were imaged live for 24 hours as described, and cultures were then fixed and immunostained for confocal analysis (described in the following).

### Immunostaining and confocal microscopy

Cultured cells were fixed in 4 % paraformaldehyde followed by 0.25 % triton incubation. Samples were then rehydrated in phosphate-buffered saline, blocked with 1 % bovine serum albumin/10 % normal goat serum, and incubated with primary antibodies for 1 hour at room temperature that included: rat anti-mouse podocalyxin (1 μg/ml; R&D Systems), mouse anti-E-cadherin (0.25 μg/ml; BD Biosciences), rabbit anti-ZO-1 (2.5 μg/ml; Invitrogen-Zymed Laboratories), mouse anti-cytokeratins (clones AE1/AE3, 1:1000; Dako), rabbit anti-ezrin (1 μg/ml; Cell Signaling Technology Inc.), and mouse anti-Muc1 (1:500; gift from Dr John Stingl, Cambridge Research Institute, Cambridge, UK)). Cells were then incubated with fluorescently conjugated species-specific secondary antibodies (Invitrogen-Molecular Probes, Burlington, ON, Canada), and nuclei were counterstained with 4′,6-diamidino-2-phenylindole (Sigma), mounted in glycerol containing the anti-fade agent diazabicyclo[2.2.2]octane (Sigma), and imaged using an Olympus FV1000 confocal microscope (Olympus) followed by processing using FV1000 Fluoview (Olympus) and Adobe Photoshop v12.0 software (Adobe, San Jose, CA, USA).

## Results

### Podocalyxin overexpression induces locally invasive collective tumor budding in vivo

**Fig. 1:**
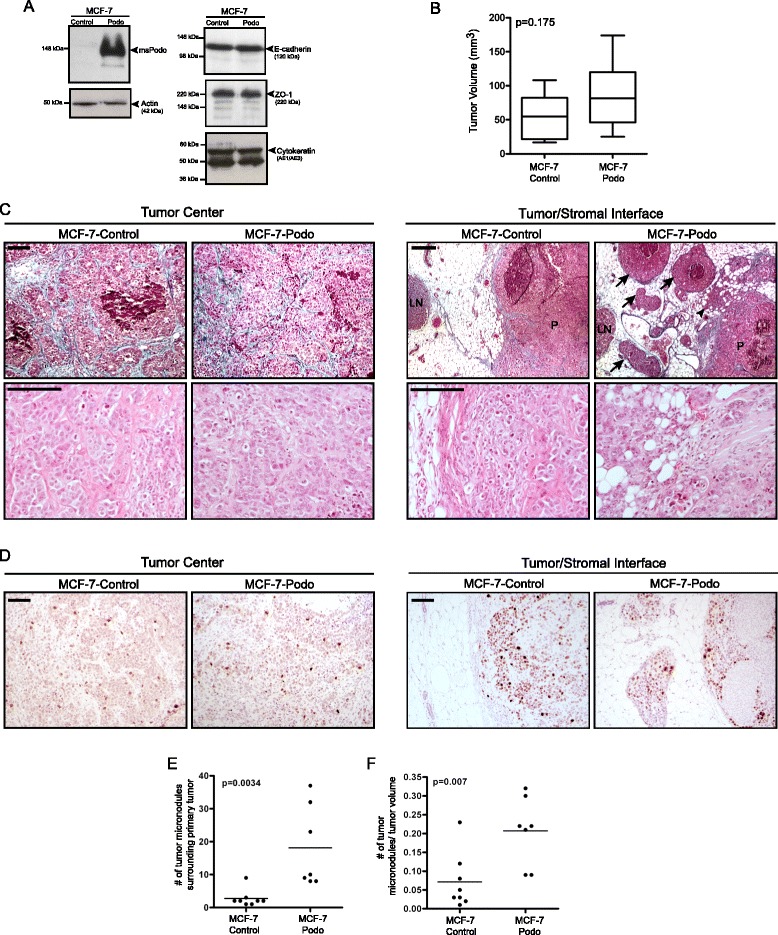
Podocalyxin overexpression induces collective tumor invasion in vivo*.* **a** Western blot analyses of whole cell lysates show that total expression of E-cadherin, the tight junction protein ZO-1, and epithelial cytokeratins were unaffected by stable podocalyxin overexpression in MCF-7-podo cells compared with MCF-7-control cells. **b** Podocalyxin overexpression did not significantly affect overall estrogen-dependent MCF-7 cell-derived tumor growth after orthotopic xenotransplantation (*n* = 8 for each condition; tumors were excised after 6 weeks; *p* = 0.175, two-tailed, unpaired Student’s *t* test). **c** Representative images of trichrome-stained tumor sections (*upper panels*) show that MCF-7-control and MCF-7-podo xenografts both contained densely packed tumor cells at the center of the primary tumor nodule. Both tumor types also had small areas of visible necrosis and developed a collagenous stroma (*light blue staining*) that surrounded nests of cohesive tumor cells. Representative images of the tumor/host stromal interfaces show that, compared with controls, MCF-7-podo cells more extensively protruded into the surrounding stroma at the edge of tumor (*arrowhead*). These tumors also formed multiple small cohesive tumor foci, or micronodular bud-like structures (*arrows*), that appeared to be separate from the primary tumor nodule (labeled *P*; local lymph node is labeled *LN*). Higher power H & E images (*lower panels*) indicate that there was no a difference in tumor cell density within either the centers or edges of the lesions at the tumor/stromal interface. Scale = 100 μm. **d** Tumors were immunostained with the proliferation marker Ki-67 (*brown*). While there was an overall increase in Ki-67-positive cells at the tumor/stromal interface, there was no discernible difference in Ki-67 frequency between MCF-7-control and MCF-7-podo cells either at the center of the tumors (*left panel*) or at the tumor/stromal interface (*right panel*). Scale = 100 μm. **e**, **f** The number of observable micronodules was quantified either per tumor **e** or per tumor volume **f**. Note that, in both cases, there was a statistically significant increase in the MCF-7-podo tumors compared with the control tumors (two-tailed, unpaired Student’s *t* test)

Human MCF-7 breast tumor cells are epithelial, form well-differentiated non-invasive tumors in vivo, and express little endogenous podocalyxin [13, 38, 40]. Thus, we generated stable MCF-7 transfectants overexpressing mouse podocalyxin (MCF-7-podo) which can be identified unambiguously in human cells using a species-specific antibody [34]. MCF-7-podo cells did not exhibit a difference in steady-state levels of the epithelial markers E-cadherin, ZO-1, or cytokeratins compared with empty vector-transfected MCF-7-control cells (Fig. 1a). Like the controls, MCF-7-podo cells also continued to form E-cadherin-containing adherens junctions in both 2-D monolayer (Fig. 3) and 3-D spheroid/aggregate (Fig. 5) culture. Therefore, podocalyxin-overexpressing MCF-7 cells remained cohesive and epithelial.

Human MCF-7-control and MCF-7-podo cells both formed palpable estrogen-dependent tumors after they were orthotopically transplanted into the mammary fat pads of immunocompromised mice. After 6 weeks there was a trend towards slightly larger tumors in the MCF-7-podo group that was not statistically significant (Fig. 1b; *p* = 0.175; *n* = 8 for each condition). The same trend towards a slight increase in tumor size in the podocalyxin overexpression condition was also observed in subcutaneously grown tumors (Additional file 1: Figure S1A). Histopathologically, the central portions of MCF-7-control and MCF-7-podo orthotopic tumors were indistinguishable; both were characterized by densely packed nests of cohesive tumor cells that were surrounded by an aniline blue-positive collagenous matrix and small areas of tissue necrosis (Fig. 1c, top panel). Furthermore, there were no distinguishable differences in cell size (Fig. 1c, bottom panel) or proliferation based on Ki67 staining (Fig. 1d, left panel) in the central portions of the tumors in either condition.

While MCF-7-control tumors formed well-defined outer borders, the outer edges of MCF-7-podo tumors were highly irregular when observed at low power (Additional file 1: Figure S2). Associated with the latter was a significantly greater number of cohesive micronodules, or buds, that surrounded the primary MCF-7-podo tumor masses (Fig. 1c, right panel, arrows; quantified in Fig. 1e, f). While Ki67 staining indicated an increase in proliferation at the tumor/stromal interface compared with the tumor center in both conditions, there was no obvious difference in Ki67 positivity in this region between the MCF-7-control and MCF-7-podo tumors at that interface (Fig. 1d, right panels).

**Fig. 2:**
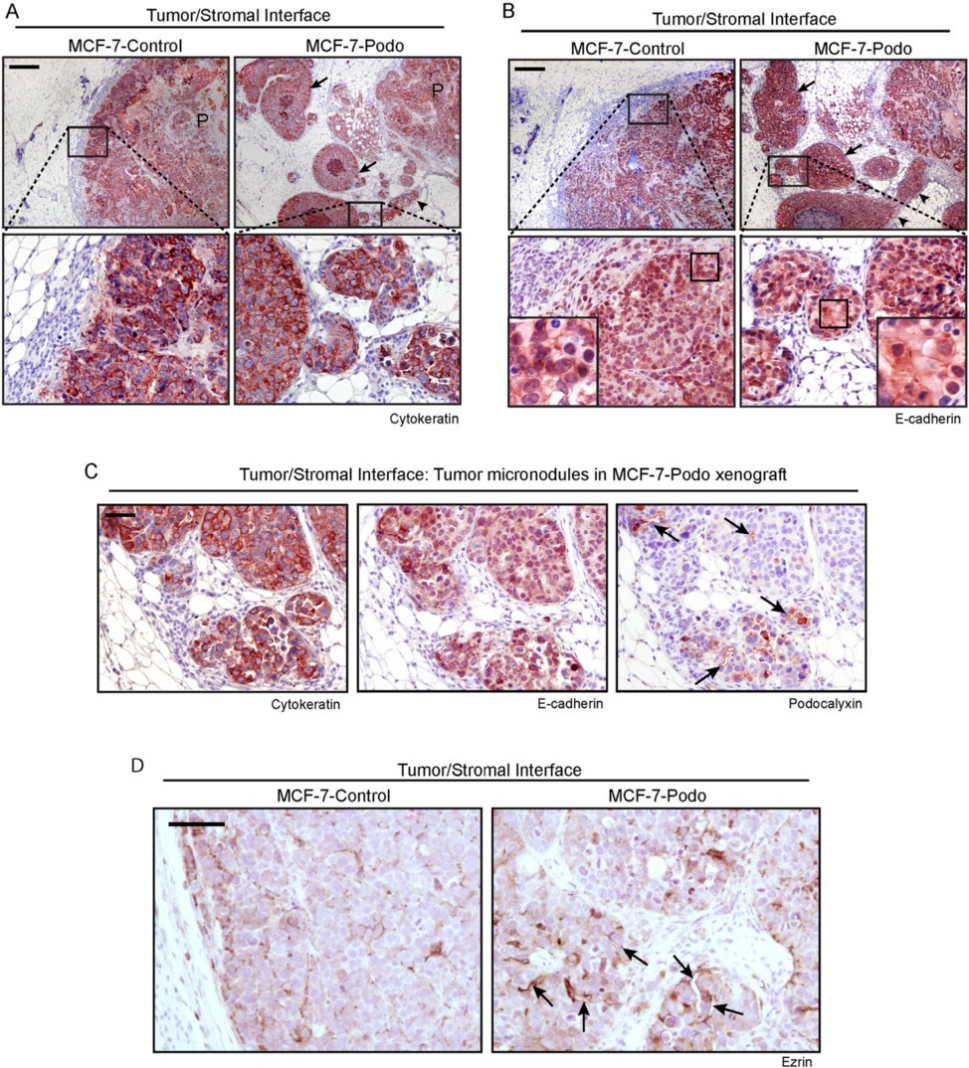
Podocalyxin overexpression induces cohesive epithelial invasion of the stroma and regional lymph node. **a**, **b** Serial sections (approximately 100 μm apart) immunostained for cytokeratin **a** and E-cadherin **b** indicated that the stromal invasion of MCF-7-podo-derived tumor micronodules was cohesive and epithelial. Notably, some cohesive micronodules were extensions originating from the main primary tumor (*arrowheads*), while others were completely disconnected from it (*arrows*) **a**, **b**. Shown in the lower panels are magnified views of the outlined areas within the upper images, with further magnified insets in the lower panels of **b** showing the presence of some membranous E-cadherin. Scale = 200 μm. **c** Representative invasive micronodules were serially sectioned and immunostained for epithelial cytokeratins, E-cadherin, and podocalyxin. Note that a considerable portion of the podocalyxin localized to small microlumenal membrane surfaces within the tumor micronodules (arrows). Scale = 50 μm. **d** MCF-7-control and MCF-7-podo tumor sections were immunostained for ezrin and the tumor/stromal interface was imaged. Note that while ezrin was often localized to cell membranes in both conditions, it also lined microlumenal structures in the MCF-7-podo tumors (arrows). Scale = 50 μm

Through a careful analysis of serial sections we determined that some of the invasive buds located at the edge of MCF-7-podo tumors were completely disconnected from the primary mass (Fig. 2a, b, arrows) while others remained connected to protrusions that emanated directly from the primary mass (Fig. 2a, b, arrowheads). The MCF-7-podo tumor micronodules were made up of densely-packed, cohesive tumor cells that, like the primary tumor, continued to express epithelial cytokeratins (Fig. 2a) and E-cadherin (Fig. 2b), some of which was membranous (Fig. 2b, lower panels, higher magnification insets). In addition, these micronodules often contained small podocalyxin-lined microlumens (Fig. 2c, arrows). Ezrin, which is an actin scaffolding protein that complexes with the cytoplasmic tail of podocalyxin [34], also lined the invasive microlumens within the invasive nodules at the tumor/stromal interface in the MCF-7-podo condition (Fig. 2d, right panel, arrows). While ezrin was also localized to the plasma membrane of tumor cells in both conditions, we observed very little evidence of ezrin-lined microlumens in the MCF-7-control condition (Fig. 2d, left panel). We concluded that podocalyxin overexpression facilitates the collective budding of invasive epithelial MCF-7 breast tumor cell micronodules into the mammary stroma in vivo and that this is associated with microlumen formation.

### Podocalyxin overexpression induces collective migration in 2-D monolayer culture

**Fig. 3:**
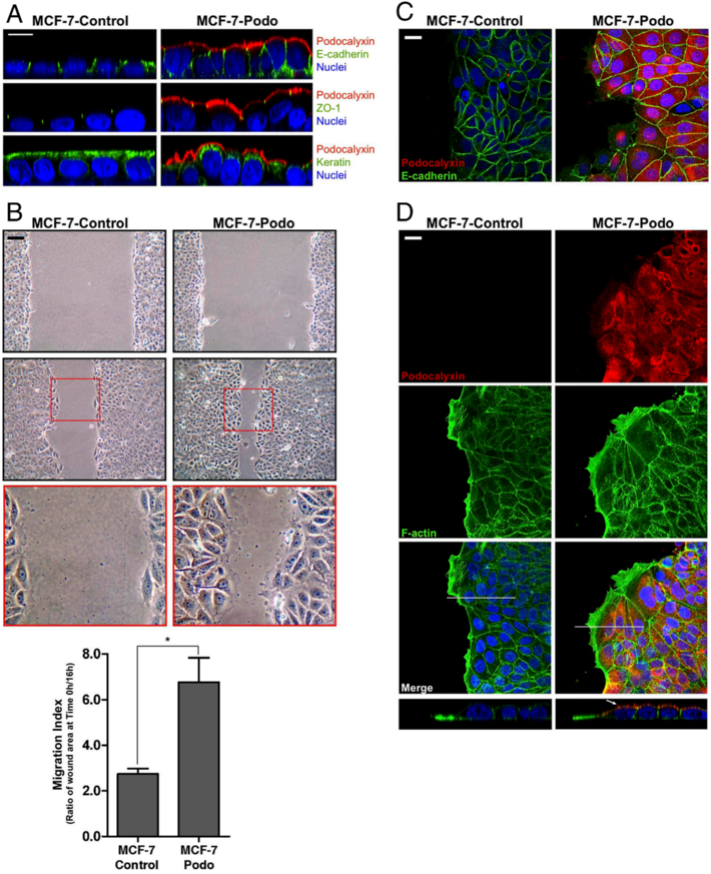
Podocalyxin stimulates collective breast tumor cell migration in 2-D monolayer culture. **a** Confocal XZ vertical images show that, as expected, podocalyxin alters the architecture of the apical membrane surface of MCF-7-podo cells maintained in 2-D culture. As a result it causes the cells to assume different shapes within the monolayers, which are much more uniform in the controls. The basolateral localization of E-cadherin is relatively unaffected in the podocalyxin-expressing cells and tight junctions are still present apically as indicated by discrete puncta of localized ZO-1, although the location of the latter varies within the vertical plane given the change in cell architecture. Podocalyxin does not cause a loss of epithelial keratin filaments but it does disrupt the uniformity of their localization at the apical surface. Scale = 10 μm. **b** Serum-starved MCF-7-control and MCF-7-podo cell monolayers attached to a rigid collagen I-coated substratum (0.25 μg/cm^2^) were subjected to a wound assay under growth factor-stimulated conditions (EGF 100 ng/ml). The ability of the cells to close the wound after 16 hours was monitored by phase microscopy, and photomicrographs of the same wound area after that period are shown (*upper panel*, 0 hours after wounding; *bottom panels*, 16 hours after wounding at low and high power). There was little difference in wound closure in non-EGF-stimulated conditions (data not shown), but the podocalyxin-expressing cells were able to close the wound more readily in response to EGF treatment compared with controls (graph, mean ± SD, unpaired Student’s *t* test, *p >0.05). Data shown are from one of three representative experiments. Scale = 50 μm. **c** MCF-7 cells were subjected to wounding as in **b** and after 16 hours they were fixed and immunostained for podocalyxin (*red*) and E-cadherin (*green*). Projections of confocal stacks at the leading edge of the wound show that podocalyxin-expressing cells continued to form adherens junctions. Scale = 10 μm. **d** MCF-7 cells were subjected to wounding as in **b**, and after 16 hours they were fixed and immunostained for podocalyxin (*red*) and f-actin (*green*). Projections of *x*/*y*-axis confocal stacks show that MCF-7-podo cells have enhanced, f-actin-rich lamellipodia at their leading migratory edges compared with MCF-7-control cells (*upper three panels*). *X–Z*-axis images (sliced along the white line shown in the *x*/*y* merged image) show that podocalyxin is polarized to the free, apical surface membrane (*arrow*), but does not extend completely into the enhanced f-actin rich lamellipodia (*lower panels*). Scale = 10 μm

Podocalyxin overexpression is known to increase the serum-induced motility of individual MCF-7 cells in 2-D monolayer culture [38]. However, when MCF-7-podo cell monolayers were maintained at confluency they remained cohesive and, while there was an expansion of the apical domain, the cells continued to form E-cadherin-containing adherens and ZO-1-containing tight junctions and they still expressed epithelial cytokeratins (Fig. 3a). When confluent monolayers were scratched and stimulated with EGF, MCF-7-podo cells at the scratch edge migrated collectively into the wound more rapidly than did MCF-7-control cells (Fig. 3b). It is unlikely that proliferation contributed significantly to the increased wound filling by the MCF-7-podo cells as the increased collective migration was observable within the first 16 hours after the monolayers were scratched. In addition, there was no significant difference in the proliferation of MCF-7-control and MCF-7-podo cells in monolayer culture (Additional file 1: Figure S1B).

MCF-7-podo cells retained their E-cadherin and actin-containing adherens junctions during collective migration (Fig. 3c, d). Interestingly, the f-actin-containing anterior lamellipodia were more prominent at the leading edges of the migrating MCF-7-podo cell sheets than were those formed by MCF-7-control cell sheets (Fig. 3b, lower panel, note phase dark lamellipodia in the MCF-7-podo cells at the wound edge; Fig. 3d, note increased f-actin in the lamellipodia of MCF-7-podo cells at the wound edge). Interestingly, podocalyxin was concentrated apically just behind these anterior lamellipodia where it colocalized with a less prominent pool of nonlamellar apical f-actin (Fig. 3d, bottom panel, *z* axis, arrow). This suggests that the demonstrated ability of podocalyxin to segregate membrane domains in an actin cytoskeleton-dependent manner [19, 34] may play a role in its ability to stimulate collective tumor cell migration.

**Fig. 4:**
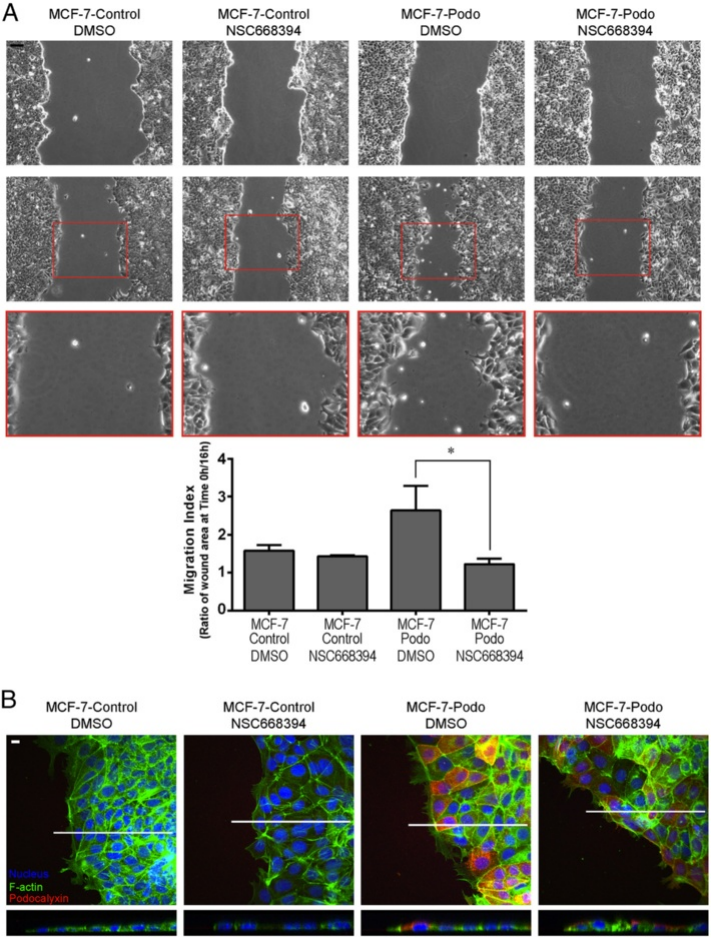
The ezrin inhibitor NSC668394 decreases collective migration and leading lamellipodia formation of podocalyxin-overexpressing cells. **a** Serum-starved MCF-7-control and MCF-7-podo cell monolayers were cultured and scratched as described in Fig. 3 in the presence of DMSO (vehicle control) or the ezrin inhibitor NSC668394, and they were imaged by phase microscopy after 16 hours. Note that NSC668394 significantly decreased the collective migration of the MCF-7-podo cells into the wound as quantified using the migration index described in Fig. 3 (mean ± SD, unpaired Student’s *t* test, *p >0.05). **b** MCF-7-control and MCF-7-podo cells were subjected to wounding in the absence and presence of NSC668394 for 16 hours. The cells were then fixed and immunostained for podocalyxin (*red*) and f-actin (*green*). Projections of *x/y* confocal stacks (*upper panels*) and *X–Z* plane images (*lower panels*) indicated that NSC668394 decreased the formation of f-actin-containing leading lamellipodia in the MCF-7-podo cells. Scale = 10 μm. *DMSO* dimethylsulfoxide

Podocalyxin interacts with the actin cytoskeleton via ezrin which binds to its cytoplasmic domain [34] and the separate interaction of ezrin with actin requires it to be phosphorylated in its “ERM” domain. When we treated MCF-7-podo cells with a pharmacological inhibitor of this phosphorylation, NSC668394 [39], there was a significant loss of the small punctate accumulations of podocalxyin and pERM at the cell surface (Additional file 1: Figure S3) which we have previously shown to be associated with microvilli in the apical domain of MCF-7-podo cell monolayers [34]. Importantly, treatment with NSC668394 also decreased the collective migration and the enhanced wound edge lamellipodia formation of scratched MCF-7-podo cell monolayers (Fig. 4).

### Podocalyxin overexpression induces collective epithelial invasion and a bud-like phenotype in 3-D culture

**Fig. 5:**
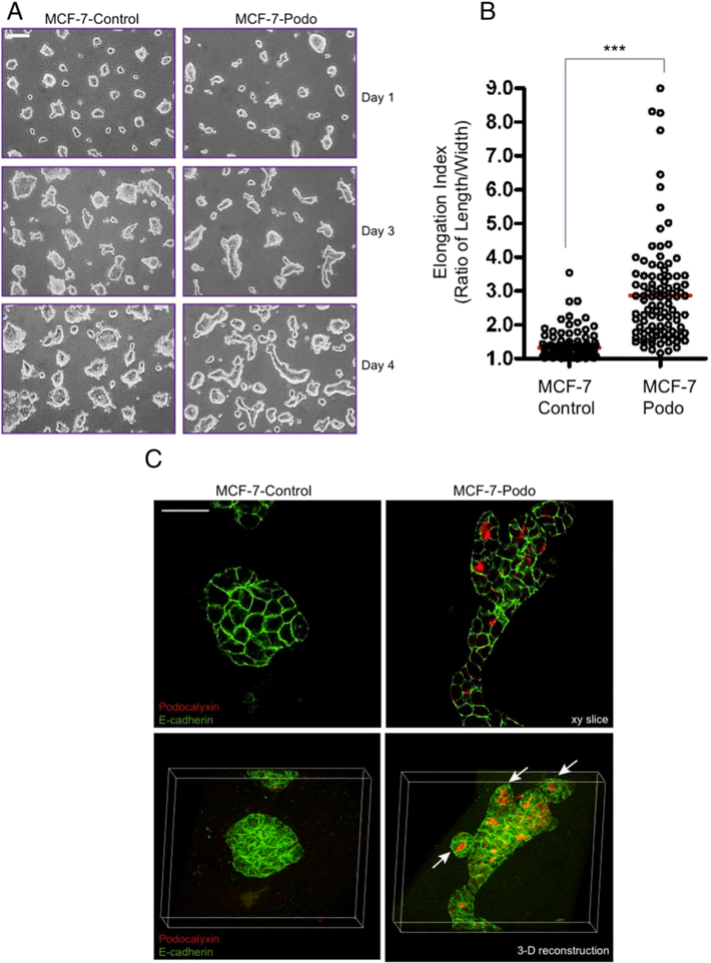
Podocalyxin stimulates collective breast tumor cell invasion in 3-D spheroid culture. MCF-7 cells were forced to form aggregates in 3-D culture by first plating them overnight on Matrigel. The aggregates were then overlaid with type I collagen in the presence of EGF for the times indicated. **a** The MCF-7-control cell aggregates grew considerably in size between day 1 and day 4 and remained relatively spherical. The MCF-7-podo cell aggregates also grew in size but, in addition, they became elongated as they protruded and collectively pushed into the matrix. Live phase microscopy; scale bar = 100 μm. **b** The collective invasion of the aggregates was quantified after 4 days in 3-D culture by determining their “elongation index”, which consisted of the longest length to width ratio for each aggregate. ***p <0.001, unpaired Student’s *t* test. **c** Cell aggregates maintained for 4 days in 3-D culture were fixed and coimmunostained for podocalyxin (*red*) and E-cadherin (*green*). Single *x*/*y*-axis confocal images (*top panels*; scale = 40 μm) and 3-D reconstructed images (*bottom panels*) are shown. The reconstructions show that podocalyxin promotes the cohesive elongation of the aggregates without disrupting E-cadherin localization at cell–cell adhesions. Note also that podocalyxin primarily localized at multiple microlumina within cohesive tumor cell bud-like structures that bulged out from the elongated cell aggregates (*arrows*). In the lower panels the white outlined box indicates the rotation of the reconstructed confocal stack; the long axis of each box is 211 μm in length

MCF-7 cells cluster together to form non-invasive aggregates when they interact with a reconstituted basement membrane ECM (i.e., Matrigel) in 3-D culture [41]. Thus, we pre-clustered MCF-7 cell populations on Matrigel and then overlaid them with collagen type I because we noted an accumulation of stromal collagen in the MCF-7 cell-derived tumors that developed orthotopically within the mammary fat pads in vivo (see Fig. 1). In addition, other investigators have shown that the presence of stromal collagen facilitates collective breast tumor cell invasion [11, 12]. Under these conditions, the MCF-7-control cell aggregates gradually increased in size over a 4-day period. Importantly, the control cell aggregates remained relatively spherical throughout (Fig. 5a, left panel). The latter characteristic was quantified by determining the elongation index (longest length/shortest width of the cell clusters where a perfect sphere has an index of 1.00) which was 1.32 ± 0.04 at the end of the experiment (Fig. 5b). In contrast, while they also started out as small spheroidal aggregates, over the 4-day culture period many of the MCF-7-podo cell clusters elongated and pushed out into the matrix as cohesive multicellular extensions with blunt-ended tips (Fig. 5a, right panel) As a result, the mean elongation index of the MCF-7-podo cell clusters was significantly larger than the controls (2.89 ± 0.1, *p* <0.001 vs. controls; Fig. 5b). The dynamic nature of this podocalyxin-mediated increase in collective tumor cell invasion into the ECM was observable by live video phase microscopy (compare Additional file 2: Movie S1 for MCF-7-control cell clusters with Additional file 2: Movie S2 for MCF-7-podo cell clusters).

MCF-7-control and MCF-7-podo cell clusters both maintained their E-cadherin-containing adherens junctions in 3-D culture (Fig. 5c). However, unlike normal mammary epithelial cells expressing podocalyxin (Additional file 1: Figure S4), MCF-7-podo cell aggregates did not fully polarize to form a single large, central lumen. Instead, the elongated MCF-7-podo cell clusters often formed multiple small podocalyxin-lined microlumens (Fig. 5c, right panel). Interestingly, the cells arranged around some of these microlumens formed small bud-like structures that were readily apparent when we generated 3-D reconstructions of the cell aggregates (Fig. 5c, lower right panel, arrows).

**Fig. 6:**
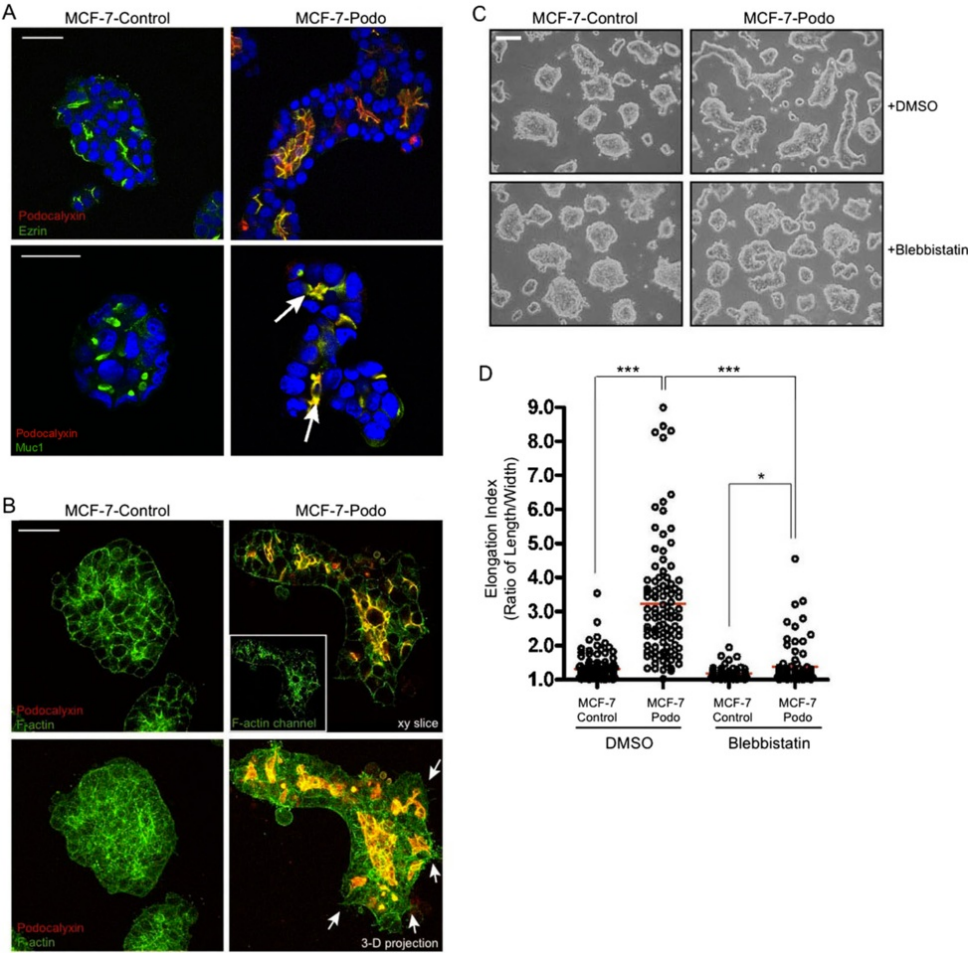
Podocalyxin-mediated collective invasion is actomyosin contractility dependent. **a** MCF-7-control and MCF-7-podo cell aggregates overlaid with collagen were fixed and immunostained for exogenous mouse podocalyxin (*red*) and either the actin cytoskeletal linker ezrin (*green*, *upper panel*) or the apical membrane marker Muc1 (*green*, *lower panel*). Single *x*/*y*-axis confocal images show that MCF-7-control cells localize ezrin to basolateral surfaces and small membranous pockets within the spheroid interior. Apical Muc1 localized to small, closed internal domains in the MCF-control cell aggregates. In contrast, MCF-7-podo cells predominantly localized ezrin and Muc1 to expanded microluminal surfaces within the elongated invasive aggregates. Scale = 20 μm. **b** MCF-7-control and MCF-7-podo cell aggregates were fixed and stained for exogenous mouse podocalyxin (*red*) and f-actin (*green*). Single *x*/*y*-axis confocal micrographs (*top panel*) and 3-D projected images (*bottom panel*) show an enrichment of f-actin along the expanded microlumenal surfaces within the MCF-7-podo cell aggregates where it colocalized with podocalyxin. Also, the outer cell surfaces of the podocalyxin aggregates exhibited prominent f-actin-rich, but podocalyxin-spare, membrane projections that probed the surrounding ECM (*arrows*). Scale bar = 40 μm. **c**, **d** MCF-7 cell aggregates were maintained in the presence of either DMSO (vehicle control) or the myosin II inhibitor blebbistatin (10 μM) for 4 days. Phase contrast images show that blebbistatin significantly blocked MCF-7-podo cells from collectively invading the matrix by elongation **c**. This is quantified in **d**. ***p <0.001, *p <0.05, Student’s *t* test; scale bar = 100 μm. *DMSO* dimethylsulfoxide

As already noted, we previously demonstrated that podocalyxin recruits the actin-scaffolding protein ezrin to the free apical surface of MCF-7 cells maintained as monolayers in 2-D culture [34]. Herein we found that ezrin co-localized with podocalyxin around the internal microlumens that formed in the collectively invasive MCF-7-podo cell aggregates in 3-D culture (Fig. 6a, upper panels). While the control cell clusters contained closed internal apical membrane domains that were marked by the mucin Muc1 (Fig. 6a, lower left panel), podocalyxin expression caused these internal domains to expand and open up as microlumens (Fig. 6a, lower right panel, arrows) that preferentially recruited f-actin to them (Fig. 6b). Interestingly, 3-D reconstructions revealed that the outer surface of MCF-7-podo cell clusters, but not MCF-7-control cell clusters, often formed f-actin-rich protrusions that were actually podocalyxin-negative (i.e., similar to the situation in the enhanced lamellipodia-like structures in 2-D monolayers) which appeared to probe the surrounding collagen matrix (Fig. 6b, bottom right panel, arrows). Very dynamic peripheral protrusions were also observed at the edge of the collectively invading MCF-7-podo cell aggregates by live video microscopy (Additional file 2: Movie S2). Thus, contractile actin-mediated protrusion formation may contribute to the collective invasion of the MCF-7-podo cells, a tentative conclusion that was supported by the finding that blebbistatin, a pharmacologic inhibitor of actomyosin-dependent contractility, greatly diminished their elongation/collective invasion (Fig. 6c, d).

### Podocalyxin depletion inhibits collective invasion in 3-D culture

**Fig. 7:**
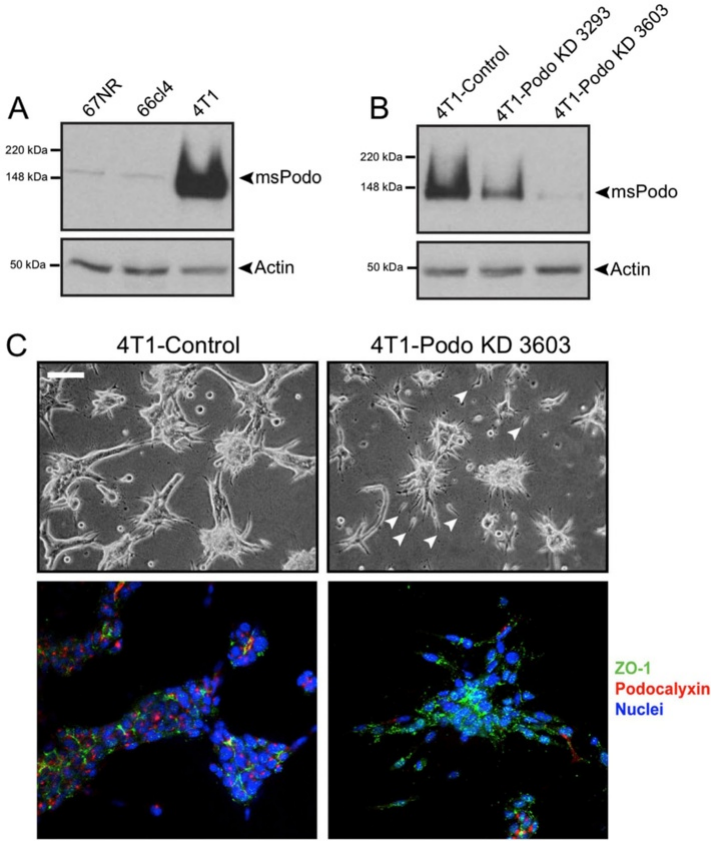
Stable knockdown of podocalyxin in highly invasive 4T1 murine breast tumor cells reduces collective invasion. **a** Whole cell lysates from syngeneic murine breast tumor cell lines with different capacities to metastasize were probed for endogenous podocalyxin expression. Endogenous podocalyxin was most highly expressed in 4T1 cells, which are highly metastatic compared with the nonmetastatic 67NR and weakly metastatic 66 cl4 cell lines [42]. **b** 4T1 cells stably transfected with either control vector (4T1-control) or vectors containing an shRNA sequence targeting the 3′ untranslated region of the murine podocalyxin transcript were analyzed for endogenous podocalyxin expression by western blotting. Stable 4T1 cell populations, each expressing a different short hairpin sequence, show a moderate (podo KD-3293) and near complete knockdown of endogenous podocalyxin (podo KD-3603), respectively. **c** 4T1-control or 4T1-podo KD 3603 tumor cells were aggregated on Matrigel overnight, overlaid with collagen type I, and maintained in 3-D culture for 4 days. They were then imaged, live, by phase microscopy (*upper panels*) or fixed and immunostained for endogenous podocalyxin (*red*) and the tight junction protein ZO-1 (*green*) followed by confocal imaging (*lower panels*). The 4T1-control cells invaded as cohesive multicellular strands with scattered small pockets of poorly polarized podocalyxin throughout. In contrast, aggregates from podocalyxin knockdown cells did not effectively invade as cohesive multicellular strands; they did, however, occasionally release single cells into the matrix (*arrowheads*). Scale = 50 μm

The highly metastatic mouse mammary tumor 4T1 cell line expresses significant amounts of endogenous podocalyxin compared with genetically similar mammary tumor cell lines that are either weakly metastatic (66 cl4) or nonmetastatic (67NR; Fig. 7a). Despite their high metastatic potential, 4T1 cells form epithelial E-cadherin-containing adherens junctions [42]. We thus reasoned that these cells were good candidates to be collectively invasive in 3-D culture, which turned out to be the case. Specifically, when we preclustered 4T1-control cells and overlaid them with collagen type 1, the clusters sent out collective, bud-like protrusions into the matrix that contained polarity-disrupted aggregations of podocalyxin, some of which appeared to be associated with microlumenal structures (Fig. 7c, left panels; Additional file 3: Movie S3). In contrast, when we stably knocked down podocalyxin, the resulting 4T1-podo KD 3603 cells (Fig. 7b) did not productively invade the matrix as cohesive cell cohorts. Instead, a small proportion of 4T1-podo KD cells moved into the gel singly in an elongated fashion (Fig. 7c, right panels, arrowheads; Additional file 3: Movie S4).

## Discussion

Podocalyxin was initially described as the major glomerular anion in the kidney that plays a critical role in the formation of the small gaps between the individual processes of podocytes that become the slit diaphragms which are critical for plasma filtration during the initial phase of urine formation [14]. This was confirmed in podocalyxin knockout mice where the glomerular epithelium remains patent and no filtration occurs [15]. Podocalyxin was later shown to be gp135, which had long been used as a marker of the apical membrane domain of MDCK kidney epithelial cells in 2-D monolayer culture [16]. Functional experiments in MDCK cells demonstrated that podocalyxin contributes to the formation of a preapical membrane domain during the formation of a single central lumen within polarized MDCK cell spheroids maintained in 3-D collagen gels [16]. Interestingly, when apical–basal polarity is actively disrupted by blocking integrin function, podocalyxin is not trafficked properly, a single central lumen does not form efficiently, and the otherwise normal MDCK epithelial cell spheroids start to move collectively through the collagen matrix [19].

When we force-expressed podocalyxin in normal mammary epithelial cells and placed the cells in 3-D culture, they clustered together and, similar to MDCK cells, formed polarized spheroids with a large, single lumen that was centrally located (Additional file 1: Figure S3). In contrast, epithelial MCF-7 breast tumor cells have an intrinsic polarity defect such that control MCF-7 spheroids did not generate a single central lumen. Instead, as demonstrated by Muc1 staining, the control MCF-7 cell clusters formed multiple small internal apical membrane domains. Forced podocalyxin overexpression in MCF-7 cells served to facilitate the expansion and separation of these domains during microlumen formation within elongating MCF-7-podo cell clusters that began to protrude collectively into the ECM. Therefore, the intrinsic polarity defect in the MCF-7 spheroids, which has been shown to be mediated, at least in part, by aberrant integrin signaling [41], may have contributed to the ability of podocalyxin to initiate their collective invasion in a fashion analogous to that which occurred in integrin-blocked MDCK cell spheroids [19].

In MDCK cells, the movement of podocalyxin to apical and luminal membrane domains that occurs during the normal polarization process is dependent on the interaction of its cytoplasmic domain with the actin scaffolding proteins ezrin, NHERF-1, and NHERF-2 [19]. We showed here that ezrin colocalized with podocalyxin at the microlumenal membranes of collectively invasive MCF-7 cell aggregates, and we have shown previously that NHERF-1 also colocalizes with podocalyxin in these cells in small punctate microvillus-like structures when they are maintained in 2-D monolayer culture [34]. Mostov and colleagues have suggested that an inappropriate targeting of podocalyxin and these associated scaffolding proteins contributes to the formation of leading lamella-like structures that drive collective MDCK cell invasion under polarity-disrupted conditions in 3-D culture [19]. We found that force-expressed apical podocalyxin localized directly adjacent to expanded actin-rich lamella at the free edge of collective migrating MCF-7 monolayers in 2-D culture. Importantly, when we treated these cells with a pharmacologic inhibitor of ezrin activation, we observed a disruption of ezrin and podocalyxin localization in the apical domain of these cells and a decrease in collective migration which broadly agrees with the findings of Mostov and colleagues who used normal epithelial cells where polarity was actively disrupted in 3-D culture to initiate an ezrin-dependent podocalyxin-mediated collective invasion [19].

We also found that podocalyxin overexpression stimulated the formation of actin-rich lamellipodia-like protrusions that emanated from the collectively invading MCF-7-podo cell aggregates in 3-D culture. Thus, podocalyxin likely acts in subtly different ways from podoplanin, which is another small mucin that initiates collective tumor cell motility by inducing the formation of filopodial rather than lamella-like structures [43]. Regardless, the ability of polarity-disrupted podocalyxin to initiate the formation of cellular processes that protrude into the matrix is likely critical for facilitating collective invasion because such processes are prominent in “leader” [11] and “trailblazer” [12] cells that drive collective invasion by heterogeneous breast cancer cell populations. Additionally, as is the case with leader and trailblazer cell-driven collective invasion, MCF-7-podo cells only generated these processes and became collectively invasive when stromal collagen (type I) was added to the 3-D culture system (i.e., they did not form processes and they were not invasive in Matrigel-only 3-D cultures; data not shown).

Podocalyxin has been shown to activate the actin nucleator cortactin in a NHERF-1-dependent manner [44]. This activation may also contribute to podocalyxin’s ability to initiate leading lamellipodial protrusions under conditions that favor collective cell motility. Additionally, podocalyxin-mediated alterations in NHERF protein localization may act to augment growth factor-mediated signaling that contributes to the collective motile phenotype given the ability of NHERF proteins to interact with multiple hormone and growth factor receptors [45]. Indeed, we found that podocalyxin overexpression increased EGF-dependent mitogen-activated protein kinase and phosphoinositide 3-kinase-dependent signaling in MCF-7 cells (Additional file 1: Figure S5). We are now manipulating the interactions of NHERF-1 (MCF-7 cells do not express appreciable amounts of NHERF-2) with the cytoplasmic tail of podocalyxin to determine which of these interactions influences EGF-dependent signaling, lamellipodial protrusion, and/or collective motility in the presence or absence of ezrin. Finally, the heavily glycosylated extracellular domain of podocalyxin could act to augment a glycocalyx-mediated stimulation of integrin-dependent signaling to further facilitate epithelial tumor cell motility [46].

A polarity protein that is linked to the cytoskeleton, podocalyxin may modulate subcellular asymmetries in cytoskeletal contractility that have also been shown to contribute to experimental collective cell motility [10]. In accordance with this, it has been demonstrated that podocalyxin augments contractile RhoA activation at the free surface of kidney epithelial cells in an ezrin-dependent manner [47, 48] and we have shown that podocalyxin-induced collective invasion is dependent on acto-myosin contractility. Sahai and colleagues have demonstrated that the Par3 and Par6 polarity proteins generate contractile asymmetries in collectively migrating squamous carcinoma cells through their interaction with the collagen-binding Discoidin Domain Receptor 1 (DDR1) [49]. Interestingly, the expression of DDR1 helps to clinically distinguish invasive ductal breast carcinomas, which are often cohesive and epithelial, from invasive lobular breast carcinoma which often generate mesenchymal cells that move through the stromal matrix in a “single file” arrangement [50]. It is intriguing to consider the possibility that podocalyxin and DDR1 may be mechanistically linked given that their expression is coordinately suppressed by miR-199b-5p, a microRNA whose loss leads to the elevation of both DDR1 and podocalyxin in acute myeloid leukemia [51].

Tumor budding, which is regarded to be an important component of local tumor cell dissemination, has significant prognostic significance in a number of solid tumor types, including breast cancer [6–8]. By the strictest definition, tumor buds are small (i.e., less than five cells [9]). Interestingly, however, larger invasive cell clusters and micronodules can have as great, or greater, prognostic significance. The latter structures, which can consist of dozens of cells, have been classified as “poorly differentiated clusters” (PDCs) in invasive breast cancer given that their polarity is often disrupted such that they do not contain single, well-defined central lumens [8]. Based on morphology, size, and lack of a single central lumen, it would appear that the collectively invasive cell clusters that we observed in MCF-7-podo cell xenotransplants more closely resemble clinical PDCs. Whether or not increases in proliferation rates play a role in this budding is not clear clinically [7–9], and while proliferating cells were present in the budding clusters of MCF-7-podo cells in the xenotransplants, any proliferative differences with control tumors were, at best, minimal. Thus, it is unlikely that proliferation is a major driver of podocalyxin-mediated collective invasion in vivo. Additionally, differences in proliferation rates were not a factor in the increased collective motility of the MCF-7-podo cells in culture.

Like smaller tumor buds, the presence of PDCs is well correlated with LVI which is another prognostic indicator of progression in invasive breast cancer [8]. Initially, we found that podocalyxin overexpression is also a prognostic indicator of poor outcome, overall, in invasive breast cancer [13]. This was later confirmed by Andrulis and colleagues, who demonstrated that podocalyxin overexpression is also positively correlated with LVI amongst an entire invasive breast cancer cohort [31]. Interestingly, however, Andrulis’ group also found that podocalyxin overexpression was negatively correlated with outcome within the LVI breast cancer subcohort itself. This suggests to us that podocalyxin may contribute to an initial local dissemination via collective tumor invasion but that a subsequent retention of podocalyxin could suppress a later more aggressive spread by helping to prevent, for example, a switch from a collective to a more mesenchymal mode of invasion. Given this, it will be interesting to determine whether endogenous podocalyxin contributes to the tightly regulated collective invasion that helps to drive branching morphogenesis during normal mammary gland development [52].

There is one report that podocalyxin facilitates a transforming growth factor beta-mediated mesenchymal transformation of A549 lung carcinoma cells [53]. However, we found that podocalyxin overexpression did not prevent cell–cell junction formation or downregulate epithelial marker expression in MCF-7 cells. We also force-expressed podocalyxin in epithelial ovarian carcinoma cells [30] and saw no evidence that this leads to the emergence of an overt mesenchymal phenotype on its own. However, we have not fully investigated the possibility that podocalyxin upregulates selected mesenchymal markers within otherwise epithelial tumor cells which have been shown to be a part of the collectively invasive breast tumor budding phenotype [9, 12]. Interestingly, when we knocked down endogenous podocalyxin in highly metastatic, but still epithelial, 4T1 mammary tumor cells, while there was a clear decrease in collective invasion there was also an increase in the movement of single elongated cells into the matrix. Thus, it is possible that a late-stage loss of podocalyxin may stimulate a shift towards a single cell mode of motility which could be associated with the emergence of a mesenchymal phenotype. Under some conditions, such a mode shift could potentially lead to the emergence of a more aggressive phenotype, a notion that is supported by the clinical data of Andrulis and colleagues already described [31]. Whether or not the loss of podocalyxin can initiate a switch between collective and mesenchymal modes of tumor cell invasion is something we are actively investigating.

In addition to invasive breast cancer, podocalyxin overexpression is also correlated with poor outcome in other epithelial-derived cancers where alterations in polarity figure prominently in tumor formation and progression. These include renal cell, colorectal, high-grade serous ovarian, and bladder carcinomas [28–30, 32, 33]. In the latter case, increased podocalyxin levels are also predictive of outcome [33]. Given these findings, we developed antibodies against the podocalyxin extracellular domain in an effort to block its tumor progression promoting function. Our initial studies indicate that this is feasible in preclinical transplantation experiments using metastatic breast and mammary tumor cell lines that overexpress podocalyxin [54]. Therefore, podocalyxin overexpression may represent a novel therapeutic target in metastatic carcinoma progression.

## Conclusions

Podocalyxin overexpression causes epithelial tumor cells to become collectively motile in culture and to initiate collectively invasive tumor budding in orthotopic transplants in vivo. Therefore, in addition to being prognostically significant for breast cancer outcome, podocalyxin overexpression may functionally contribute to breast cancer progression.

## Additional files

Additional file 1: Is Figure S1 showing podocalyxin has little effect on subcutaneous tumor size **a** or proliferation in monolayer culture **b**, Figure S2 showing podocalyxin overexpression promotes local invasion of MCF-7 tumor cell xenografts,. Figure S3 showing that the ezrin inhibitor NSC668394 disrupts apical podocalyxin localization in monolayer culture, Figure S4 showing normal mammary epithelial cells continue to form spheres and form single, polarized lumens in 3-D culture, and Figure S5 showing podocalyxin expression increases EGF-mediated signaling. (ZIP 1056 kb)
Additional file 2: Is Movie S1 showing control MCF-7 cells present little collective invasion 3-D culture, and Movie S2 showing podo-overexpressing cells send out very dynamic processes and show considerable collective invasion in 3-D culture. (ZIP 2262 kb)
Additional file 3: Is Movie S3 showing collective invasion of control 4T1 cells that express considerable endogenous podocalyxin in 3-D culture, and Movie S4 showing decreased collective invasion of 4T1 cells in 3-D culture when endogenous podocalyxin is stably knocked down. (ZIP 1243 kb)

## Abbreviations

2-D: Two dimensional
3-D: Three dimensional
DDR1: Discoidin Domain Receptor 1
DMSO: Dimethylsulfoxide
ECM: Extracellular matrix
EGF: Epidermal growth factor
EMT: Epithelial to mesenchymal transition
FACS: Fluorescent-activated cell sorting
H & E: Hematoxylin and eosin
LVI: Lymphovascular invasion
PDC: Poorly differentiated cluster
shRNA: Short hairpin RNA
