# Supplementary material for: The cell surface mucin podocalyxin regulates collective breast tumor budding
Source: Breast Cancer Res. 2016 Jan 22;18:11. doi: 10.1186/s13058-015-0670-4 (PMC4722710; doi:10.1186/s13058-015-0670-4)

Figure S5: Podocalyxin expression increases EGF-mediated signaling.

MCF-7-control and MCF-7-podo cells in subconfluent monolayer culture were serum starved overnight and treated with EGF (25 ng/ml) for the indicated times. Western blot analysis of whole cell lysates indicated that podocalyxin overexpression (MCF-7-Podo) enhanced downstream p-ERK and p-AKT signaling in response to EGF stimulation compared to MCF-7-control cells.

Supplemental Figure 5

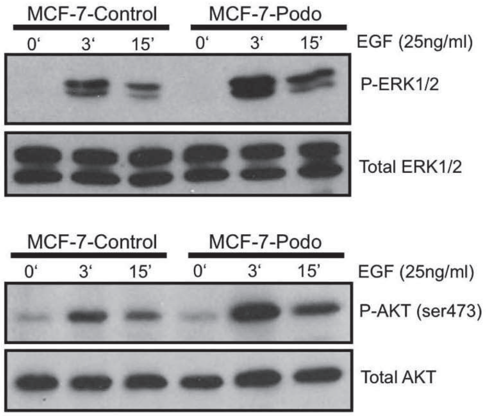

Supplement: Additional file 1: — Is Figure S1 showing podocalyxin has little effect on subcutaneous tumor size a or proliferation in monolayer culture b, Figure S2 showing podocalyxin overexpression promotes local invasion of MCF-7 tumor cell xenografts,. Figure S3 showing that the ezrin inhibitor NSC668394 disrupts apical podocalyxin localization in monolayer culture, Figure S4 showing normal mammary epithelial cells continue to form spheres and form single, polarized lumens in 3-D culture, and Figure S5 showing podocalyxin expression increases EGF-mediated signaling. (ZIP 1056 kb) [file 13058_2015_670_MOESM1_ESM.zip › Figure S5.pdf]
