# Supplementary material for: The cell surface mucin podocalyxin regulates collective breast tumor budding
Source: Breast Cancer Res. 2016 Jan 22;18:11. doi: 10.1186/s13058-015-0670-4 (PMC4722710; doi:10.1186/s13058-015-0670-4)

Figure S4: Normal mammary epithelial cells continue to form spheres and form single, polarized lumens in 3-D culture.

Normal EpH4 mammary epithelial cells were stably transfected with a podocalyxin-containing expression vector as described in the Materials and Methods. The cells were then pre-aggregated on polyHEMA-coated plates, overlaid with Matrigel for the indicated times (Roskelley et al., 1994) and co-immunostained with the apical tight junction marker ZO-1 (green) and podocalyxin (red). Note that within 24 hours the Matrigel overlay caused the relocalization of ZO-1 and podocalyxin centrally (ie. apically) and this was followed by the formation of a single central lumen after 48 hr that enlarges through 72 hr. The same occurred when these cells were initially aggregated on Matrigel and overlaid with collagen I (not shown).

Reference:

Roskelley CD, Desprez PY, Bissell MJ: Extracellular matrix-dependent tissue-specific gene expression in mammary epithelial cells requires both physical and biochemical signal transduction. Proc Natl Acad Sci USA 1994, 91(26):12378-82.

Supplemental Figure 4

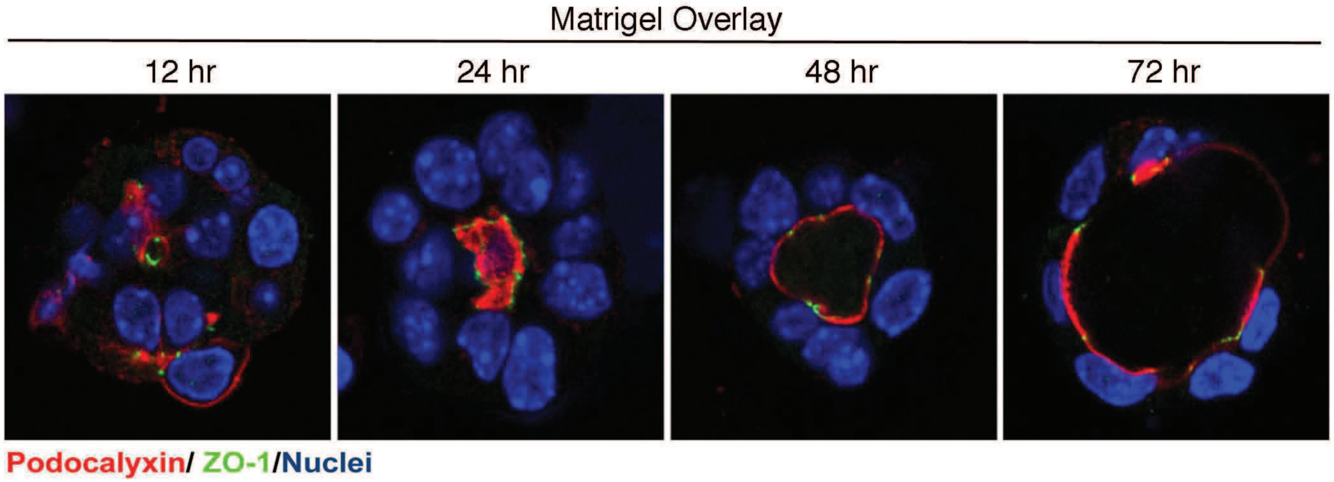

Supplement: Additional file 1: — Is Figure S1 showing podocalyxin has little effect on subcutaneous tumor size a or proliferation in monolayer culture b, Figure S2 showing podocalyxin overexpression promotes local invasion of MCF-7 tumor cell xenografts,. Figure S3 showing that the ezrin inhibitor NSC668394 disrupts apical podocalyxin localization in monolayer culture, Figure S4 showing normal mammary epithelial cells continue to form spheres and form single, polarized lumens in 3-D culture, and Figure S5 showing podocalyxin expression increases EGF-mediated signaling. (ZIP 1056 kb) [file 13058_2015_670_MOESM1_ESM.zip › Figure S4.pdf]
