# Supplementary material for: The cell surface mucin podocalyxin regulates collective breast tumor budding
Source: Breast Cancer Res. 2016 Jan 22;18:11. doi: 10.1186/s13058-015-0670-4 (PMC4722710; doi:10.1186/s13058-015-0670-4)

Figure S3: The ezrin inhibitor NSC668394 disrupts apical podocalyxin localization in monolayer culture.

MCF-7-control and MCF-7-podo cells were maintained as subconfluent monolayers in the absence (DMSO, vehicle control) or presence of the ezrin inhibitor NSC668394 for 16 hr. The cells were fixed with 4% paraformadehyde, triton-extracted to leave cytoskeletal-associated antigens in place, and immunostained for podocalyxin (red) and phosphorlyated ERM (pERM; which captures the active actin-binding form of ezrin). Note that NSC668394 treatment decreased cytoskeletal-associated pERM in both cell populations and disrupted the punctate apical cell surface podocalyxin in MCF-7-podo cells that we have previously shown is microvillar in nature (Nielsen et al., 2007).

Reference:

Nielsen JS, Graves ML, Chelliah S, Vogl AW, Roskelley CD, McNagny KM: The CD34-related molecule podocalyxin is a potent inducer of microvillus formation. PLoS ONE 2007, 2(2):e237.

Supplemental Fig 3

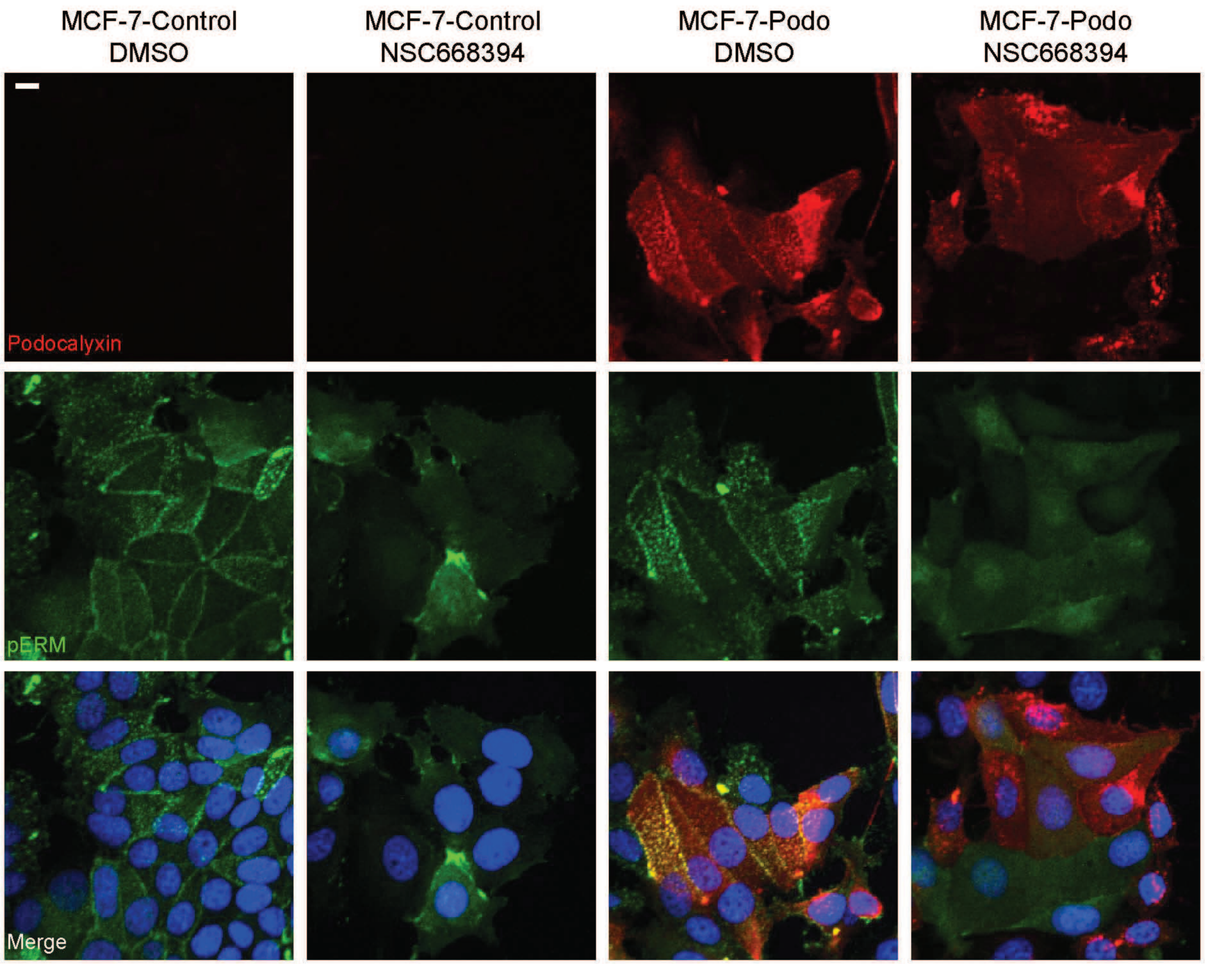

Supplement: Additional file 1: — Is Figure S1 showing podocalyxin has little effect on subcutaneous tumor size a or proliferation in monolayer culture b, Figure S2 showing podocalyxin overexpression promotes local invasion of MCF-7 tumor cell xenografts,. Figure S3 showing that the ezrin inhibitor NSC668394 disrupts apical podocalyxin localization in monolayer culture, Figure S4 showing normal mammary epithelial cells continue to form spheres and form single, polarized lumens in 3-D culture, and Figure S5 showing podocalyxin expression increases EGF-mediated signaling. (ZIP 1056 kb) [file 13058_2015_670_MOESM1_ESM.zip › Figure S3.pdf]
