# Supplementary material for: The cell surface mucin podocalyxin regulates collective breast tumor budding
Source: Breast Cancer Res. 2016 Jan 22;18:11. doi: 10.1186/s13058-015-0670-4 (PMC4722710; doi:10.1186/s13058-015-0670-4)

Figure S2: Podocalyxin overexpression promotes local invasion of MCF-7 tumor cell xenografts.

Tissue sections from three independent MCF-7-control and MCF-7-podo orthotopic xenografts were trichrome-stained and entire mammary gland sections were digitally scanned at low power to better visualize all of the primary tumor mass and any tumor nodules that formed. Compared to control, the podocalyxin expressing tumor cells formed irregular tumor borders with more stromally-invasive micronodules surrounding one central solid primary tumor nodule. The local lymph nodes, where visible, are labeled with an *.

Supplemental Fig 2

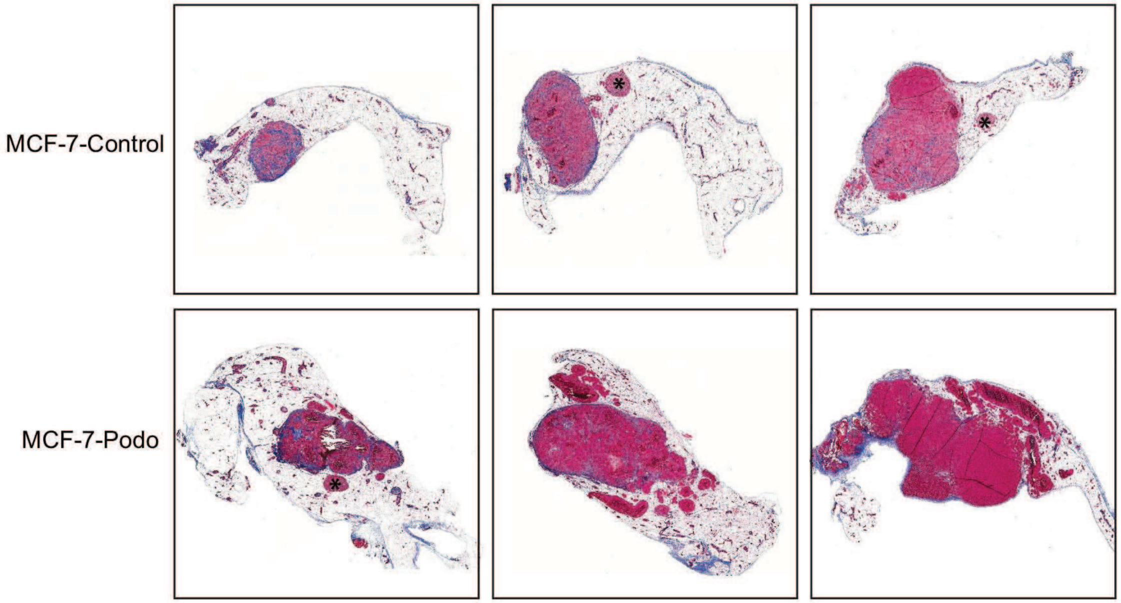

Supplement: Additional file 1: — Is Figure S1 showing podocalyxin has little effect on subcutaneous tumor size a or proliferation in monolayer culture b, Figure S2 showing podocalyxin overexpression promotes local invasion of MCF-7 tumor cell xenografts,. Figure S3 showing that the ezrin inhibitor NSC668394 disrupts apical podocalyxin localization in monolayer culture, Figure S4 showing normal mammary epithelial cells continue to form spheres and form single, polarized lumens in 3-D culture, and Figure S5 showing podocalyxin expression increases EGF-mediated signaling. (ZIP 1056 kb) [file 13058_2015_670_MOESM1_ESM.zip › Figure S2.pdf]
