# Supplementary material for: The cell surface mucin podocalyxin regulates collective breast tumor budding
Source: Breast Cancer Res. 2016 Jan 22;18:11. doi: 10.1186/s13058-015-0670-4 (PMC4722710; doi:10.1186/s13058-015-0670-4)

Figure S1: Podocalyxin has little effect on subcutaneous tumor size (A) or proliferation in monolayer culture (B)

(A): 1 X 10^7 MCF-7-control or MCF-7-podo cells were injected into the subcutaneous tissue of the flanks of 12 week old Rag 2M mice that contained slow release 17ß-estradiol pellets (see Methods and Materials for details). Tumor volume was determined after 80 days using the formula 0.52[length (mm)] x [width (mm)] x [height (mm)]. Note that there was a slight trend towards larger tumors in the MCF-7-podo condition (n=13 for each condition; p=0.0671, two-tailed, unpaired Student's t-test).

(B): Cell proliferation of MCF-7-control and MCF-7-podo cells in 2D monolayer culture was determined using an MTT colorimetric assay for cell growth and viability (Mosmann, 1983). Briefly, cells were resuspended in either serum containing or serum free regular growth media, and 5 X 10^3 cells were plated in triplicate in individual wells of a 96 well plate (one plate per timepoint). On the indicated days, cells were incubated with MTT (3-(4,5-dimethylthiazol-2-yl)-2,5-diphenyltetrazolium bromide) for 4 hours at 37°C. Cells were lysed in DMSO and the solublized reaction product was quantified using a multi-well scanning spectrophotometer. Note that there was very little discernible difference in growth rate between the two cell lines in either medium supplementation condition.

Reference:

Mossman T. Rapid colorimetric assay for cellular growth and survival: application ot proliferation and cytoxicity assays. J Immunol 1983, 65:55-63.

Supplemental Fig 1

A

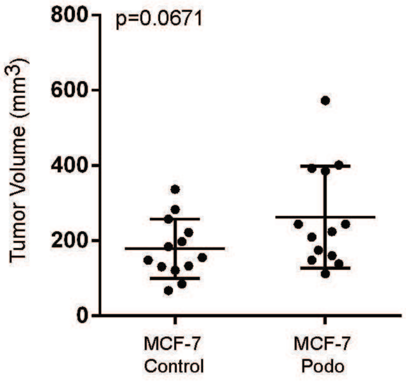

B

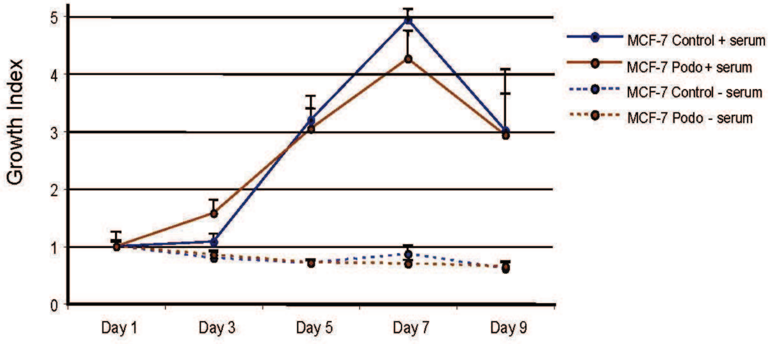

Supplement: Additional file 1: — Is Figure S1 showing podocalyxin has little effect on subcutaneous tumor size a or proliferation in monolayer culture b, Figure S2 showing podocalyxin overexpression promotes local invasion of MCF-7 tumor cell xenografts,. Figure S3 showing that the ezrin inhibitor NSC668394 disrupts apical podocalyxin localization in monolayer culture, Figure S4 showing normal mammary epithelial cells continue to form spheres and form single, polarized lumens in 3-D culture, and Figure S5 showing podocalyxin expression increases EGF-mediated signaling. (ZIP 1056 kb) [file 13058_2015_670_MOESM1_ESM.zip › Figure S1.pdf]
