# Supplementary material for: The cell surface mucin podocalyxin regulates collective breast tumor budding
Source: Breast Cancer Res. 2016 Jan 22;18:11. doi: 10.1186/s13058-015-0670-4 (PMC4722710; doi:10.1186/s13058-015-0670-4)
Supplement: Additional file 2: — Is Movie S1 showing control MCF-7 cells present little collective invasion 3-D culture, and Movie S2 showing podo-overexpressing cells send out very dynamic processes and show considerable collective invasion in 3-D culture. (ZIP 2262 kb) [file 13058_2015_670_MOESM2_ESM.zip › Movies S1 and S2 caption.doc]

**Movies S1 and S2:**

Time-lapse videos of collagen I-overlaid MCF-7-control (Movie S1) and MCF-7-podo (Movie S2) cell aggregates on Matrigel (see Materials and Methods for details) were imaged, live, by phase microscopy for a 24-hour period. The overlaid aggregates of MCF-7-control cells (Movie S1) were somewhat contractile but remained mostly spherical throughout. In contrast, the overlaid MCF-7-podo cell aggregates (Movie S2) were highly contractile, sent out many dynamic processes into the matrix, and they elongated and pushed into the matrix collectively.

During acquisition, phase contrast images were captured every 5 minutes. To reduce file size, videos were generated by extracting every second frame eg. (every 10 minutes) using Leica AF6000 software. The files were then digitally compressed using Adobe Media encoder software.
