# Supplementary material for: The cell surface mucin podocalyxin regulates collective breast tumor budding
Source: Breast Cancer Res. 2016 Jan 22;18:11. doi: 10.1186/s13058-015-0670-4 (PMC4722710; doi:10.1186/s13058-015-0670-4)
Supplement: Additional file 3: — Is Movie S3 showing collective invasion of control 4T1 cells that express considerable endogenous podocalyxin in 3-D culture, and Movie S4 showing decreased collective invasion of 4T1 cells in 3-D culture when endogenous podocalyxin is stably knocked down. (ZIP 1243 kb) [file 13058_2015_670_MOESM3_ESM.zip › Movies S3 and S4 caption.doc]

**Movies S3 and S4:**

Time-lapse videos of collagen I-overlaid 4T1-control (Movie S3) and 4T1-podo KD (Movie S4) cell aggregates on Matrigel (see Materials and Methods for details) wereimaged live, by phase microscopy, over a 24-hour period. The overlaid aggregates of 4T1-control cells (Movie S3) elongated and moved into the surrounding collagen matrix as cohesive multicellular cohorts. In contrast, aggregates from podocalyxin knockdown cells (Movie S4) did not effectively invade collectively. Instead, single cells were observed radiating from the aggregates, dynamically probing the matrix and were occasionally seen to detach from the cluster completely and invade singly.

Phase contrast images were captured, extracted and compressed as described for Supplemental Movies 1 & 2. Please note that Supplemental Movie 3 reveals a technical anomaly that occurred during image acquisition at 3hr:08min:52sec in which images were captured in succession (for approximately 1 minute) until 3hr:09min:09sec and then resumes at a frequency of 20 minutes per frame. Despite this, the overall acquisition time indicates a duration of ~24 hours comparable to what is shown in Supplemental Movie 4.
